# Influence of printing orientation on the accuracy, fit and mechanical properties of 3D-printed full-coverage prostheses: a systematic review

**DOI:** 10.3389/froh.2026.1789942

**Published:** 2026-08-11

**Authors:** Passent Ellakany, Nourhan M. Aly, Rania Moussa, Hassan F. Albrahim, Abdulrahman Al Saadoun, Sadeem Alzaghran, Chin-Chuan Fu, Gilan Y. Altonbary

**Affiliations:** 1 Department of Restorative Sciences, School of Dentistry, The University of Alabama at Birmingham, Birmingham, AL, United States; 2 Department of Pediatric Dentistry and Dental Public Health, Faculty of Dentistry, Alexandria University, Alexandria, Egypt; 3 Department of Substitutive Dental Sciences, College of Dentistry, Taibah University, Al-Madinah Al-Munawarah, Saudi Arabia; 4 College of Dentistry, Imam Abdulrahman Bin Faisal University, Dammam, Saudi Arabia; 5 Department of Substitutive Dental Sciences, College of Dentistry, Imam Abdulrahman Bin Faisal University, Dammam, Saudi Arabia; 6 Department of Removable Prosthodontics, Faculty of Dentistry, Mansoura University, Mansoura, Egypt

**Keywords:** accuracy, dental prosthesis, marginal fit, mechanical properties, printing orientation

## Abstract

**Aim:**

This systematic review evaluated the influence of 3D printing orientation on the mechanical properties, accuracy, and marginal fit of full-coverage dental prostheses.

**Materials and methods:**

A comprehensive search was conducted through PubMed, Embase, Scopus, and Web of Science databases for *in vitro* studies published between January 2014 and June 2024. Following PRISMA 2020 guidelines based on a predefined PICO framework, *in vitro* studies assessed the printing orientation of 3D-printed full-coverage dental prostheses over the past ten years and published in English were included. Data extraction focused on printing orientation, technology, evaluation methods, and outcomes.

**Results:**

About 20 studies were included. Printing orientation significantly affected mechanical properties, including flexural and compressive strength, particularly in vertically or obliquely oriented specimens, whereas microhardness, density, and porosity were less influenced. Trueness and precision were significantly improved by printing orientation angles such as 0°, 30°, and 45°, while the greatest deviations were reported at 45°, depending on prosthesis type, tooth morphology, and printer system. Marginal fit was highly dependent on experimental conditions, with no universally optimal printing angle identified.

**Conclusion:**

Overall, printing orientation plays a critical role in the functional and dimensional performance of 3D-printed dental prostheses. Oblique orientations generally provide a favorable balance between mechanical strength and accuracy.

## Graphical Abstract

**Figure:**
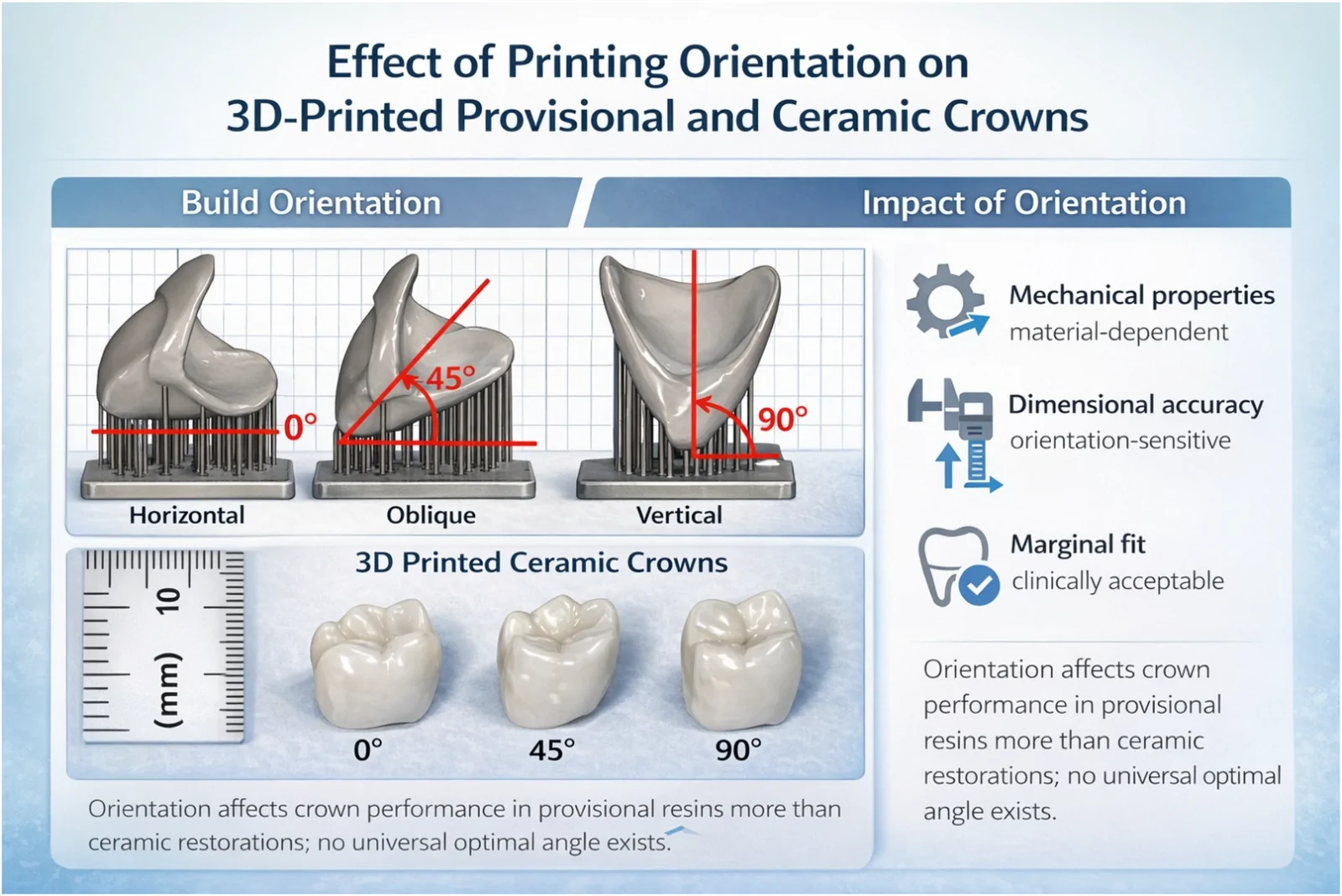

## 1 Introduction

Digital full-coverage prostheses are fundamental to modern dental rehabilitation. They are fabricated using Computer-Aided Design/Computer Aided Manufacturing (CAD/CAM) technologies through either subtractive (milling) or additive (3D printing) methods. Milling involves carving the dental prosthesis from pre-polymerized discs or blocks to the desired shape, which leads to significant material waste, tool wear, and an inability to reproduce undercuts, limiting its application in complex geometries (1).

In contrast, 3D printing fabricates prostheses by layering material to create the final design, allowing for faster production and minimal material waste (1). It enables the creation of customized restorations with complex geometries and undercuts that are difficult to achieve through milling, enhancing its applicability in patient-specific treatments (2). Additionally, 3D-printed prostheses demonstrate acceptable mechanical and aesthetic properties, as well as a cost-effective alternative for restorative and prosthetic dentistry in terms of both initial investment and production expenses (3). However, current limitations of 3D printing include the need for post-processing procedures to achieve the desired surface finish, which may compromise the dimensional stability and mechanical properties of the final prosthesis (4).

Various 3D printing technologies are currently utilized in prosthodontics, each utilizing different operating principles and resin materials. Stereolithography (SLA), for example, uses a laser beam to cure photosensitive liquid resin layer by layer, providing high accuracy and a smooth surface finish (5). Digital Light Processing (DLP) is a vat-photopolymerization 3D printing technology that cures photosensitive resin layers using projected ultraviolet (UV) light, offering faster printing speeds compared to other methods (6). Fused Deposition Modeling (FDM) is an additive manufacturing technique that extrudes thermoplastic material through a heated nozzle, building the object layer by layer according to digital design data (7). While FDM may yield fixed dental prostheses (FDPs) with lower accuracy and increased surface roughness compared to conventional methods, it remains a cost-effective option particularly suitable for producing prototypes, dental models, and applications involving educational or low-demand clinical use (7).

Selective Laser Sintering (SLS) is an advanced 3D printing technology that fabricates precise and durable dental components by using a high-powered laser to fuse powdered materials such as polymers, metals, ceramics, and composites, layer by layer. It is particularly beneficial for producing metallic frameworks where high mechanical performance is essential (8). Other 3D printing technologies used in dentistry include Direct Metal Laser Sintering (DMLS), Selective Laser Melting (SLM), Multi-Jet Fusion (MJF), and several emerging additive manufacturing methods (9).

The printing technology used plays a critical role in determining the resolution and surface quality of dental restorations (10). The quality of materials used in 3D printing significantly influences the overall manufacturing outcome. Key parameters such as printing technology, layer thickness, build orientation, light type, temperature conditions, and post-curing methods collectively impact the mechanical and dimensional properties of the final structure. Among these, printing orientation has emerged as a particularly critical factor affecting both accuracy and performance (11–13).

Selecting an appropriate printing orientation can enhance performance by minimizing contact with critical regions of the printed model. The object can be oriented at any angle between 0° and 359°, typically categorized as horizontal (*X*-axis), vertical (*Y*-axis), or oblique (angles between the X and Y axes). Each orientation affects the number of layers and support structures required, thereby influencing the mechanical properties and overall quality of the additively manufactured prosthesis (13).

Although several reviews have examined building orientation in dental printing, most focused on single material classes (e.g., provisional resins) or single printing technologies (e.g., SLA only). Moreover, none have incorporated the rapidly expanding evidence base from 2023 to 2024, particularly recent studies on ceramic-SLA zirconia and hybrid resin-ceramic materials. Existing reviews therefore lack a comprehensive, up-to-date synthesis across materials specifically for full-coverage prostheses, which differ fundamentally from printed models, dentures, or splints.

The present review fills this gap by integrating the most recent literature and providing a structured, material-specific analysis of how printing orientation affects mechanical properties, accuracy (trueness and precision), and marginal adaptation of 3D-printed full-coverage prostheses. Rather than seeking a universal optimal angle, this review identifies material-dependent patterns and offers clinically relevant recommendations for selecting orientation based on restoration and printing technology. The null hypothesis states that printing orientation does not affect the accuracy, mechanical properties and marginal fit of 3D-printed dental restorations.

## 2 Materials and methods

The protocol of this systematic review was registered in the International Prospective Register of Systematic Reviews (PROSPERO, No.: CRD42024547829). Any changes from the initial protocol are detailed in this manuscript.

### 2.1 Search strategy and data acquisition

The search strategy and data acquisition followed the Preferred Reporting Items for Systematic Reviews and Meta-Analyses (PRISMA) guidelines.

**Study Outcomes (PICOS):** The search question was structured following the PICOS framework: **Population (P):** Full-coverage dental prostheses. **Intervention (I):** 3D printing manufacturing technique. **Comparator (C):** Dental prostheses printed at different printing angles. **Outcome (O):** Mechanical properties, accuracy (trueness and precision), and marginal fit of the dental prostheses. The research question is: What is the best printing orientation to achieve optimal mechanical properties and accuracy in fabricating full-coverage dental prostheses?

Studies were identified by searching electronic databases of PubMed, Web of Science, Scopus and Embase. The keywords applied to the search were: (“crown” OR “dental prostheses” OR “implant supported” OR “dental restoration” OR “full-coverage” AND (“printing, three dimensional” OR “ 3D print” OR “selective laser melting” OR “Additive manufacture” OR “CAD” OR “CAM” OR “Computer-aided design” OR “Computer-aided manufacture” OR “Stereolithography” OR “Digital technology” OR “Digital manufacture”) AND (“Accuracy” OR “Fit” OR “Precision” OR “Adaptation” OR “Trueness” OR “Strength” OR “Hardness” OR “Mechanical properties” OR “Surface characteristics” OR “Color stability”) NOT(REVIEW OR MEDICAL OR REPORT).

Search terms were tailored for each database to account for variations in indexing practices and search syntax. The detailed search results for each database are as follows; **PubMed:** before Filtering (initial search): 10/6/2024–2742, After Filtering (last 10 years, only English, not systemic): 10/6/2024–1785, After Duplicate Removal: 10/8/2024–1476, After Filter by Title and Abstract: 10/8/2024–463 and after Filter by Full Text: 3/9/2024–24. **Embase:** Before Filtering (initial search): 10/6/2024–300, After Filtering (last 10 years, only English, not systemic): 10/6/2024–241, after Duplicate Removal: 10/8/2024–149, After Filter by Title and Abstract: 10/8/2024–463, after Filter by Full Text: 3/9/2024–24. **Web of Science:** Before Filtering (initial search): 10/6/2024–3847, after Filtering (last 10 years, only English, not systemic, dental and biomaterial field): 10/6/2024–1115, after Duplicate Removal: 10/8/2024–987, After Filter by Title and Abstract: 10/8/2024–463 and after Filter by Full Text: 3/9/2024–24. And for **Scopus:** before Filtering (initial search): 13/6/2024–4262, after Filtering (only English, not systemic): 13/6/2024–4037, after Duplicate Removal: 10/8/2024–2424, after Filter by Title and Abstract: 10/8/2024–463 and after Filter by Full Text: 3/9/2024–24.

Additionally, to expand the search, a manual search of gray literature sources such as the Cochrane Library Trials, ClinicalTrials.gov registry, ProQuest, and Google Scholar, along with the references of included studies, was performed.

The inclusion criteria were peer-reviewed studies published in English investigating 3D-printed full-coverage dental prostheses oriented at various build-up angles, with clear documentation of the printing angles. Only *in vitro* or laboratory studies published in English language during the last decade (January 2014 to June 2024) were included. Excluded studies were those not relevant to the research (PICO) question, non-comparative studies, reviews, case reports, and clinical studies. In addition to research papers with non-extractable data or investigating printed materials for other dental, medical, or industrial applications as well as those published in other language than English.

Screening process started in June 2024, two reviewers (H.F. and A.S.) independently screened the electronic databases. The web search results were downloaded to the reference manager software (http://rayyan.qcri.org). Duplicated records were eliminated. Articles were initially screened through the titles and abstracts based on the predefined eligibility criteria followed by screening of full texts for eligibility. Any disagreements were resolved through discussion; if consensus could not be reached, a third reviewer (P.E.) was consulted to resolve the conflict. Inter-rater reliability was assessed using Cohen's kappa coefficient (*κ*).

For Data Extraction, two independent reviewers (P.E. and G.A.) extracted and tabulated the data from each relevant article. A custom data extraction sheet was created, and the following details were collected from all the included studies: publication details, including (authors and year of publication); aim; total sample size; study groups; methodological details, including the 3D printing orientation, 3D printer, milling machine and method of conventional fabrication (if applicable); evaluation outcomes; and conclusions. Because the included studies used varying definitions for printing angles and axis references, the present review standardized terminology as follows and presented in Table with the advantages and limitations of the mentioned angles (Table 1):

- 0° = object oriented horizontally (parallel to the build platform)
- 90° = object oriented vertically (perpendicular to the build platform)
- Oblique angles (e.g., 30°, 45°, 60°, 120°, 135°) = angles between horizontal and vertical orientations

When studies reported anatomical descriptors (e.g., “occlusal surface facing platform,” “margin facing platform”), the description is retained, and the equivalent angle is clarified in parentheses when provided by authors. The current review offers a descriptive analysis of the studies included, considering the methodological details of the materials and the assessment methods used.

**Table 1:** Summary of the influence of build orientation on the mechanical properties, dimensional accuracy, and fit of 3D-printed full-coverage dental prostheses.

Orientation	Potential advantages	Potential limitations
0° (Horizontal)	Improved trueness in several studies; reduced layer-induced anisotropy	Increased support contact area; additional finishing may be required
45° (Oblique)	Often provides balanced accuracy and mechanical performance; reduced stair-stepping effect	Results highly dependent on material and restoration geometry
90° (Vertical)	Improved compressive strength in some studies; reduced support contact on certain surfaces	Greater anisotropic behavior; lower trueness reported in several studies

### 2.2 Risk of bias analysis

A risk of bias analysis was performed on the included studies, following PRISMA guidelines to ensure transparency and reproducibility in the current systematic review. The modified CONSORT statement was utilized to evaluate the included studies. Thirteen items were applied for the risk of bias judgment, where the item that was fulfilled was recorded “yes”, and if not fulfilled as “No”. The total percentage of votes differentiated the risk of bias into high (≥ 80%), moderate (80-50), or low (< 50%) quality (3, 14).

A meta-analysis was not feasible due to extreme heterogeneity in materials, printing technologies, orientation definitions, layer thicknesses, evaluation methods, and outcome reporting metrics (e.g., RMS deviation vs. µm dimensional change vs. flexural strength). Fewer than three studies reported comparable quantitative data for any single combination of material and outcome, which violates the methodological requirements for pooled analysis.

## 3 Results

**Figure 1:**
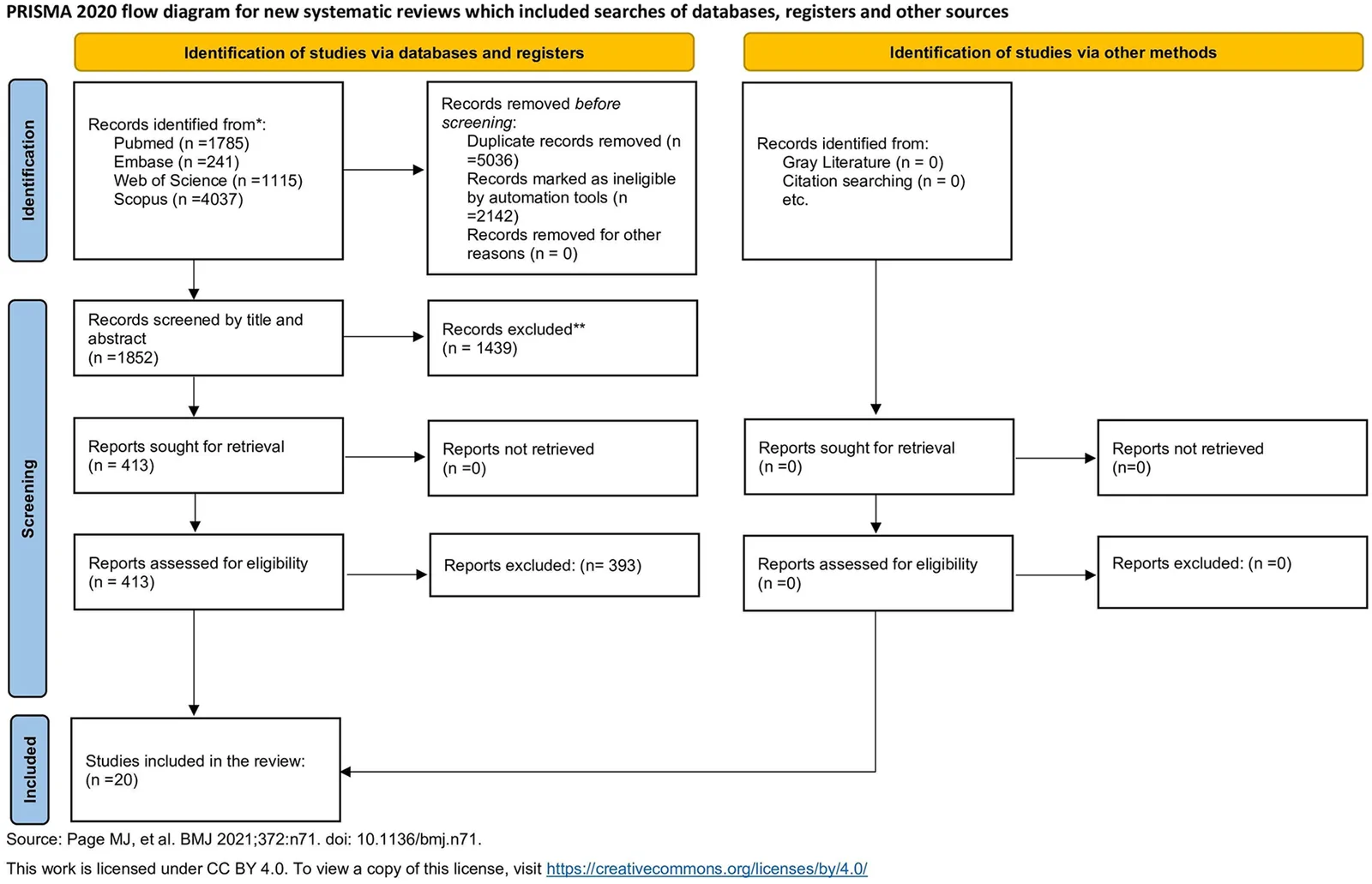
Prisma 2020 flow diagram of the study selection process.

Following PRISMA guidelines, 20 studies met the eligibility criteria and were included (Figure 1). The characteristics of the studies are summarized in Table 2. Findings are presented by material category to account for the heterogeneity in resin, hybrid ceramic, and ceramic-SLA systems.

**Table 2:** Characteristics of the included studies.

Author and year	Aim	Study groups	3D printing orientation	3D printer	Milling machine	Conventional method	Conclusion
Diken Turksayar et al, 2022 (2)	Evaluate the effect of printing orientation on the fracture strength of 3-unit interim full-coverage dental prostheses fabricated by additive and subtractive manufacturing after thermomechanical aging​	Five 3D printing orientations: 0°, 30°, 45°, 90°, 150° Control group: Subtractive manufactured (milled PMMA) (*N* = 60, *n* = 10/ group)	0° 30° 45° 90° 150°	Form 3 SLA printer	CEREC MC X5	N/A	Printing orientation significantly affected the fracture strength. Specimens printed at 0° exhibited similar fracture strength to milled PMMA. Printing at 90° resulted in the lowest strength, making those specimens more susceptible to fracture under occlusal forces​
Tahayeri et al, 2018 (4)	Compare the mechanical properties of stereolithography 3D printed provisional crowns and bridge restorations against those of conventional cures.	3D-printed specimens (using different resin settings and printing orientations) and conventionally cured materials	0° 15° 45° 90°.	FormLabs1 + stereolithography 3D printer	N/A	Integrity® Jet®	Printing orientation had an insignificant effect on length error % while the % error in width was the lowest at 90°, followed by 0, 15, and 45°. Thickness accuracy was highest at 0° and was significantly lower at 15°, 45°, and 90°. The elastic modulus of 90° printed was significantly lower than Integrity and comparable to Jet, while its Peak stress was comparable to Integrity and higher than Jet.
Dehurtevent et al, 2021 (5)	Evaluate effect of different build orientations on physical and mechanical properties, and microstructure of SLA alumina dental ceramics	Three printing orientation groups (*n* = 30/group)	ZX ZY XY	CryoCeram Printer	N/A	N/A	Density and Vickers hardness were similar across all groups. Weibull moduli were higher for ZX- and ZY-oriented specimens. ZY-oriented specimens had higher 3-point flexural strength and fracture toughness. The ZY orientation produced a reliable dental ceramic by SLA.
Farkas et al, 2023 (6)	Investigate the effect of printing layer thickness and orientation on the mechanical properties of a DLP 3D-printed dental resin.	Groups based on printing orientations (0°, 45°, 90°) and layer thickness (0.05 mm and 0.1 mm). (*N* = 36, tensile=24 and compressive = 12 samples)	0° 45° 90°	ANYCUBIC Photon Mono X (DLP 3D printer).	N/A	N/A.	Thinner layers (0.05 mm) had higher tensile strength Specimens printed at 90° showed the highest compression strength. 45° had the highest deviation from the original model dimensions.
Dikova et al, 2018 (7)	Compare the dimensional accuracy and surface roughness of polymeric dental bridges manufactured using three different 3D printing technologies.	Three types of 3D printing systems were evaluated: Digital Light Processing (DLP) Stereolithography (SLA) Laser-assisted SLA Fused Deposition Modeling (FDM) (*N* = 14)	Vertical Horizontal	RapidShape D30 (DLP SLA) Form 1+ (Laser-assisted SLA) Leapfrog Creatr Dual Extruder (FDM)	N/A	N/A	SLA and DLP produced printed objects larger than the basic model, and FDM produced smaller ones and the highest roughness. Large deviation was on the Z- and XZ-axis.
Lee et al, 2023 (10)	Measure the trueness of zirconia crowns manufactured using stereolithography (SLA) 3D printing.	Two groups BM and. BO based on the printing orientation of the crown (*N* = 20, *n* = 10/group)	BM: Margin facing the printing platform. BO: Occlusal surface facing the print platform.	CERAMAKER 900, a stereolithography (SLA) printer.	N/A	N/A	Internal trueness of BO was significantly better than BM, the same for the marginal trueness. More defects were detected for the BM group internally and at the margins. Orienting the occlusal surface towards the build platform during SLA printing improves accuracy and minimizes errors.
Revilla-León et al, 2025 (11)	To assess the influence of different print orientations on the intaglio surface accuracy (trueness and precision) of tilting stereolithography (TSLA) definitive resin-ceramic crowns	Four groups of different printing orientation (*N* = 120, *n* = 30/group)	0° 45° 75° 90°	DFAB Chairside	N/A.	N/A	The 0° orientation resulted in the best trueness and precision. The 90-degree orientation had the worst values.
Yang et al. 2022 (12)	Analyze the marginal fit of three-unit resin prostheses printed using the stereolithography (SLA) method with different build orientations and layer thicknesses	Group 1: 45° and 50 µm layer thickness Group 2: 45° and 100 µm layer thickness Group 3: 60° and 50 µm layer thickness Group 4: 60° and 100 µm layer thickness (*N* = 40, *n* = 10/group)	45° 60°	SLA 3D printer	N/A	N/A	The 45° orientation providing a better fit than the 60° orientation. The layer thickness did not have a significant effect on the marginal fit
Alharbi et al, 2016 (15)	Evaluate the effect of build direction on the mechanical properties of a novel 3D-printed dental restorative material	Two groups (*N* = 40, *n* = 20/group)	Vertical Horizontal	DW028D 3D printer (SLA technique)​	N/A	N/A	Vertically printed specimens had significantly higher compressive strength (297 MPa) than horizontally printed specimens (257 MPa).
Nold et al, 2021 (16)	Compare the fracture resistance of milled versus additive-manufactured three-unit (FPDs) and bar-shaped specimens.	Bar-shaped: Milled (*n* = 8) 3D printed (*n* = 24 (8/orientation). FPD: milled (*n* = 16) 3D printing orientations: *n* = 80, (16/group). Half dynamically loaded and aged, and half statically loaded.	Bars: Vertical Diagonal Horizontal FPD: Occlusal (O) Vertical (V) Palatal (P) Diagonal (D 45◦)	SLA printer Form2	five-axis milling machine	N/A	The vertically oriented were higher than the diagonally and horizontally printed. In non-loaded FPDS, the 3D-printed D group had the highest fracture capacity and P the lowest.
de Castro et al, 2022 (17)	Evaluate the effect of 3D printing build orientation on the accuracy, flexural modulus, flexural strength, and microhardness of 3D-printed provisional resins (3DRs)	Three groups based on build-up orientation: (*n* = 20/group)	0° 45° 90°	Formlabs 2 P30 Flash Forge Hunter W3D	CAM 5-S1	N/A	The 90° orientation showed the best overall accuracy for most resins except Cosmos-DLP. Build orientation had minimal impact on flexural modulus and flexural strength for most resins, except Cosmos-SLA. Microhardness was not influenced by print orientation​.
Abualsaud et al. 2022 (18)	Evaluate the physio-mechanical and surface properties of 3D-printed zirconia and compare these properties with milled zirconia.	Group: Subtractive milling. 3D-Printed Groups: (*n* = 20/group)	Horizontal (0°) Tilted (45°) Vertical (90°)	CERAMAKER C900 Flex	5- axis dry milling PrograMill PM7	N/A	No significant difference between groups for density, porosity, and microhardness. The tilted group had the highest surface roughness. The horizontal group had the highest contact angle. BFS of the milled group was significantly higher, while vertical and tilted had a similar BFS that was significantly lower than horizontal. 5. The highest Weibull modulus in tilted and lowest in milled.
Miura et al, 2023 (19)	Test the effect of the building direction on the mechanical and surface properties of additive-manufactured zirconia specimens.	Three groups based on the building directions (*n* = 10/group)	Parallel (0°) Diagonal (45°) Perpendicular (90°).	CeraMaker 900 stereolithography (SLA).	N/A	N/A	The highest flexural strength was observed in the perpendicular direction. The Vickers hardness, fracture toughness, elastic modulus, and Poisson's ratio showed no significant variation across different directions.
Espinar et al, 2024 (20)	Evaluate the effect of printing orientation on the flexural strength and elastic modulus of different 3D-printed dental restorative resins	FT (Formlabs Temporary) (Formlabs Permanent) DFT (Detax Freeprint Temp) GCT (GC Temporary) (*n* = 20/group)	0° 90°.	SLA Printer: Form 3B+ DLP Printer: Asiga Max UV	N/A	N/A	No significant difference in flexural strength or elastic modulus between 0° and 90° orientations for any resin.
Osman et al, 2017 (21)	Evaluate the effect of build orientation on the dimensional accuracy of full-coverage dental restorations using DLP-AM	Nine groups of different build-up angles	90°, 120° 135°, 150° 180°, 210° 225°, 240° 270°	Digital light-processing technology printer	N/A	N/A	The build angle significantly influenced the dimensional accuracy of 3D-printed dental restorations. A 135° angle provided the highest accuracy
Cameron et al, 2024 (22)	Investigate the effect of printing orientation on the trueness of additively manufactured zirconia crowns compared to subtractively manufactured (milled) crowns	Four groups: 3 printed groups and Milled crowns for comparison (*n* = 3/group)	0° 45° 90°	Lithoz CeraFab System S65 AON ZipPro	a 5-axis subtractive manufacturing milling machine	N/A	The 45° orientation with the Lithoz printer yielded results comparable to milled crowns, and 90° significantly differed from the milled.
Farag et al, 2024 (23)	Evaluate the effect of 3D printing technology and build orientation on the marginal accuracy of dental crowns​	Group 1: Digital Light Processing (DLP) Group 2: Stereolithography (SLA) Each group subdivided into three printing angles (*n* = 30/group)	0° 45° 90°	DLP: NexDent 5100 SLA: Form 2	N/A	N/A	SLA showed better accuracy compared to DLP Crowns printed at 0° had the smallest marginal discrepancy compared to 45° and 90° orientations.
Ryu et al. 2020 (24)	Evaluate the marginal and internal fit of 3D-printed provisional crowns fabricated at different build directions using the DLP-based 3D printing method	Six groups based on build directions were tested (*n* = 20/group)	120° 135° 150° 180° 210° 225°	DLP-based 3D printer	N/A	N/A	The best marginal and internal fit was achieved with build angles of 150° and 180°.
Yu et al, 2021 (25)	Evaluate the trueness of the intaglio surface and the margin quality of interim crowns fabricated using SLA 3D printing at various build angles.	Maxillary central incisor, first premolar, and first molar at 6 builds (*n* = 17)	90°, 120° 135°, 150° 180°, 210° 225°, 240° 270°	ZENITH U (SLA technology).	N/A	N/A	The central incisor and first premolar showed the lowest RMS value at 180°, and the first molar at 210°. The worst margin quality observed in all teeth was at 180 degrees.
Metin et al, 2024 (26)	Examine the influence of different build angles on the accuracy (trueness and precision) of 3D-printed crowns, veneers, and table-tops made from a hybrid resin-ceramic material	Groups based on fivebuild angles: 0°, 30°, 45°, 60°, and 90° (*n* = 50/restoration)	0° 30° 45° 60° 90°	Digital Light Processing (DLP)	N/A	N/A	Regarding trueness, a 30° angle appears optimal for crowns and table-tops. All build angles are acceptable for veneers, except for 90°. All restoration types exhibited the lowest trueness and precision at the 90° build angle along the z-axis. Regarding precision, 30° and 45° angles seem to be ideal for crowns and table-tops.

### 3.1 Interim/provisional resin prostheses

#### 3.1.1 Mechanical properties

Ten studies evaluated the effect of printing orientation on the mechanical behavior of provisional or resin-based full-coverage prostheses (2, 4, 6, 15–17, 20).

Flexural strength was the most frequently assessed parameter. Printing orientation significantly affected flexural strength in five studies (2, 4, 16, 20), whereas two studies found minimal or no effect (15, 17). Espinar et al. (20) reported that resin composition and printing technology (SLA vs. DLP) played a larger role in flexural and elastic modulus than orientation alone. Similarly, de Castro et al. (17) found that only specific resin formulations were sensitive to orientation.

Compressive strength was significantly influenced by the building angle in two studies (6, 15). Alharbi et al. (15) reported substantially higher compressive values for vertically (90°) printed specimens (297 MPa) compared with horizontal (0°) ones (257 MPa), likely due to reduced interlayer weaknesses. However, Farkas et al. (6) found tensile strength improved at horizontal (0°) ones with 0.05-mm layers.

Other properties including microhardness, density, fracture toughness, elastic modulus, and Poisson's ratio were consistently unaffected by orientation across multiple studies (16–18). This suggests that intrinsic resin chemistry may outweigh layer arrangement for these parameters.

Surface roughness behaved differently: roughness values varied with both resin type and technology, but orientation showed inconsistent effects. FDM-printed specimens demonstrated the highest roughness, while SLA produced smoother surfaces (7, 18).

#### 3.1.2 Marginal fit

Four studies assessed the impact of orientation on the marginal adaptation of resin provisional crowns or fixed dental prostheses (12, 23–25).

- Horizontal orientation (0°) yielded the smallest discrepancy in mandibular molar crowns (23) Oblique angles (45°, 150°, 180°) resulted in superior fit in two studies using SLA and DLP systems: Yang et al. (12), Ryu et al. (24).
- Vertical orientation (90°) produced the best marginal quality in Yu et al. (25)

The marked variability reflects differences in resin formulations, printer type, and tooth morphology. No universally optimal orientation emerged for provisional materials.

#### 3.1.3 Accuracy (trueness and precision)

Ten studies investigated the influence of orientation on dimensional accuracy (4, 6, 7, 10, 11, 17, 21, 22, 25, 26). Accuracy outcomes were highly angle dependent. Yu et al. (25) reported that trueness varied by both tooth type and build angle, with central incisors and premolars performing best at 180° (oblique), while molars favored 210° (oblique). Lee et al. (10) found improved trueness when the build orientation was vertical for SLA-printed zirconia crowns Metin et al. (26) reported optimal trueness at oblique (30°–45°) crowns, while precision was lowest at vertical ones (90°). Some studies recommended horizontal angles as low as 0° or oblique angles with specific values such as 114.5° and 135° depending on geometry and technology (21, 22).

Overall, accuracy in resin prostheses is influenced by resin viscosity, layer stacking direction, and distribution of support structures, making optimal orientation case specific.

### 3.2 Hybrid resin-ceramic and definitive resin prostheses

#### 3.2.1 Mechanical properties

Hybrid and definitive resin materials demonstrated similar orientation-dependent behavior to provisional resins, but the magnitude of changes was smaller.

Farkas et al. (6) reported that tensile and compressive behavior could improve when layers aligned with the anticipated load path. Other studies found no significant influence of orientation on microhardness or density (16, 18).

Hybrid materials appeared less sensitive to orientation than interim resins, likely due to higher filler content and reduced polymerization shrinkage.

#### 3.2.2 Marginal fit

Only two studies assessed hybrid or definitive resin crowns specifically, with conflicting results:

- Yang et al. (12) reported improved adaptation at oblique orientation with angle 45°.
- Farag et al. (23) observed better results at horizontal orientation (0°).

Due to limited reporting, no definitive trend can be established.

#### 3.2.3 Accuracy

Hybrid ceramic-like resins showed better trueness at 30°–45° in Metin et al. (26). However, other studies pointed to 0° or 90° depending on geometry (4).

Accuracy in these materials appears highly dependent on part geometry, support distribution, and resin composition.

### 3.3 Zirconia and ceramic-based additive prostheses

#### 3.3.1 Mechanical properties

Ceramic-based SLA and DLP systems are newer, and only a few studies evaluated zirconia or ceramic-reinforced printed crowns. Mechanical outcomes depended more on ceramic particle distribution than on orientation.

Dehurtevent et al. (5) noted that certain diagonal orientations (e.g., ZY configuration) yielded superior mechanical performance in ceramic-SLA specimens, possibly due to reduced stair-stepping.

#### 3.3.2 Marginal fit

One zirconia-AM study reported smoother marginal lines at 45° compared with milled zirconia (18). Another ceramic study found minimal differences across orientations.

Ceramic materials appear less sensitive to orientation in marginal adaptation than resins.

#### 3.3.3 Accuracy

Evidence from the zirconia-AM studies showed oblique orientations (e.g., 135°) improved trueness in DLP prints (21). Cameron et al. (22) found accuracy varied more by printer type than angle (industrial vs. desktop). Lee et al. (10) showed improved trueness in vertical builds for SLA zirconia crowns.

However, these findings require cautious interpretation due to limited sample sizes and early-stage AM ceramic technologies.

## 4 Discussion

This systematic review evaluated the influence of printing orientation on the mechanical properties, dimensional accuracy, and marginal fit of 3D-printed full-coverage dental prostheses fabricated using different additive manufacturing technologies. Across the included studies, printing orientation was an important parameter affecting restoration performance; however, its impact varied substantially depending on resin formulation, filler content, printing technology, and restoration geometry. Thus, the null hypothesis was rejected, as multiple studies demonstrated significant orientation-dependent variations across these outcomes.

Provisional and resin-based materials demonstrated the highest sensitivity to printing orientation, with several studies reporting improved flexural and compressive strength at vertical or oblique angles due to the anisotropic nature of photopolymerized resins (2, 4, 16, 20). However, other investigations found minimal orientation-related effects (6, 17), suggesting that resin formulation and printer type may play a greater role than build direction alone. Properties such as microhardness, density, fracture toughness, elastic modulus, and Poisson's ratio were largely unaffected by orientation (16–18), indicating dependence on material composition rather than layer arrangement. Surface roughness showed inconsistent orientation effects and was influenced more strongly by resin chemistry and printing technology, with FDM yielding the roughest surfaces and SLA producing smoother ones (7, 18).

Orientation also influenced marginal adaptation, though results differed across studies. Some reported the best marginal fit at 0° horizontal (23), while others favored oblique angles such as 45°, 150°, or 180° (12, 24), and one study found superior margins at vertical orientation with 90° (25). These variations likely reflect differences in crown anatomy, resin behavior, layer thickness, and support configuration. Nonetheless, all studies reported marginal gaps within clinically acceptable limits, indicating that while orientation can enhance fit, it is unlikely to compromise clinical performance. Dimensional accuracy encompassing trueness and precision showed the strongest dependence on orientation. Several studies recommended oblique angles, including 30°–45° (26), 114.5° (22), or 135° (21), owing to reduced distortion and improved dimensional accuracy. Conversely, others found horizontal or vertical orientations preferable depending on material characteristics and anatomical complexity (4, 10). Yu et al. (25) demonstrated that optimal orientation varied by tooth type, underscoring the need to consider anatomical geometry and support distribution when selecting print direction. These findings reinforce that no universal orientation is appropriate for all restorations; instead, orientation must be tailored to material behavior, restoration design, and printer specifications.

Hybrid resin-ceramic materials showed similar but less pronounced orientation effects compared with provisional resins, likely due to higher filler content and reduced polymerization shrinkage (6, 16, 18). Although improvements in trueness at oblique orientations have been documented (26), discrepancies among study findings emphasize the need for further focused evaluation of these materials.

In zirconia and ceramic-based AM systems, available evidence remains limited but suggests that build orientation plays a smaller role than factors such as ceramic particle dispersion, refractive behavior, and printer type (5, 10, 18, 19, 22). Some studies reported enhanced trueness or smoother margins at specific angles, while others found negligible differences, reflecting the developing nature of ceramic AM technologies.

Recent evidence published after completion of the literature search further supports the multifactorial nature of additive manufacturing outcomes. Alammar et al. (27) highlighted that the accuracy of 3D-printed fixed dental restorations is influenced by several interacting manufacturing variables, including printing technology, material composition, and processing protocols. Similarly, Yassine et al. (28) demonstrated using micro-computed tomography that restoration accuracy and fit are affected by both manufacturing technique and restorative material. Furthermore, a recent systematic review by Mosaddad et al. (29) evaluated the accuracy and fit of 3D-printed and milled monolithic zirconia crowns. Although that review focused on zirconia restorations rather than the predominantly resin-based prostheses included in the present review, and compared manufacturing workflows rather than build orientation, its findings similarly emphasize that restoration performance is influenced by multiple manufacturing-related factors. Therefore, the present review complements the existing literature by specifically evaluating the influence of build orientation on the mechanical properties, dimensional accuracy, and fit of 3D-printed full-coverage prostheses across different material systems.

Across material categories, several consistent trends were observed. No single printing orientation optimized all outcomes because performance depended on material composition, restoration geometry, and printing technology. Resin-based prostheses were the most sensitive to orientation, while hybrid and ceramic materials showed less dependency. Dimensional accuracy was particularly affected by support design, layer thickness, and printer type, contributing to heterogeneity across studies. Despite these variations, most mechanical, accuracy, and marginal fit values remained clinically acceptable, indicating that orientation optimization enhances quality but must be interpreted within the context of material and design considerations.

From a clinical perspective, the selection of build orientation should be individualized according to the restoration type, material system, and additive manufacturing technology employed. Although oblique orientations frequently demonstrated favorable outcomes, no universally optimal orientation could be identified because performance remains dependent on the interaction between manufacturing variables.

This review also revealed important limitations in the current evidence. The available evidence is derived predominantly from *in vitro* investigations. Definitions of printing orientation were inconsistent across studies, and variations in layer thickness, resin chemistry, light intensity, and post-curing contributed to substantial heterogeneity. Additionally, the absence of thermomechanical aging limited the applicability of findings to long-term clinical performance. Future studies should standardize orientation terminology, incorporate clinically relevant aging protocols, and conduct material- and technology-specific investigations to develop reliable guidelines for additive manufacturing in dentistry.

## 5 Conclusion

Printing orientation influences the mechanical properties, accuracy, and marginal fit of 3D-printed full-coverage dental prostheses, with the greatest effects observed in provisional resin materials. No single orientation consistently performed best across all materials or printing technologies. Importantly, all evaluated orientations produced restorations within clinically acceptable limits, indicating that orientation choice is unlikely to compromise clinical outcomes when proper protocols are used. Clinicians should therefore tailor printing orientation to the material, restoration design, and printer characteristics rather than relying on a universal recommendation.
